# Supplementary material for: Pharmacologic Activation of Integrated Stress Response Kinases Inhibits Pathologic Mitochondrial Fragmentation
Source: bioRxiv. 2024 Nov 18:2024.06.10.598126. Originally published 2024 Jun 10. Preprint. [Version 2] doi: 10.1101/2024.06.10.598126 (PMC11195119; doi:10.1101/2024.06.10.598126)
Supplement: Supplement 1 [file NIHPP2024.06.10.598126v2-supplement-1.pdf]

bioRxiv preprint doi: https://doi.org/10.1101/2024.06.10.598126; this version posted November 18, 2024. The copyright holder for this preprint (which was not certified by peer review) is the author/funder, who has granted bioRxiv a license to display the preprint in perpetuity. It is made available under aCC-BY 4.0 International license.

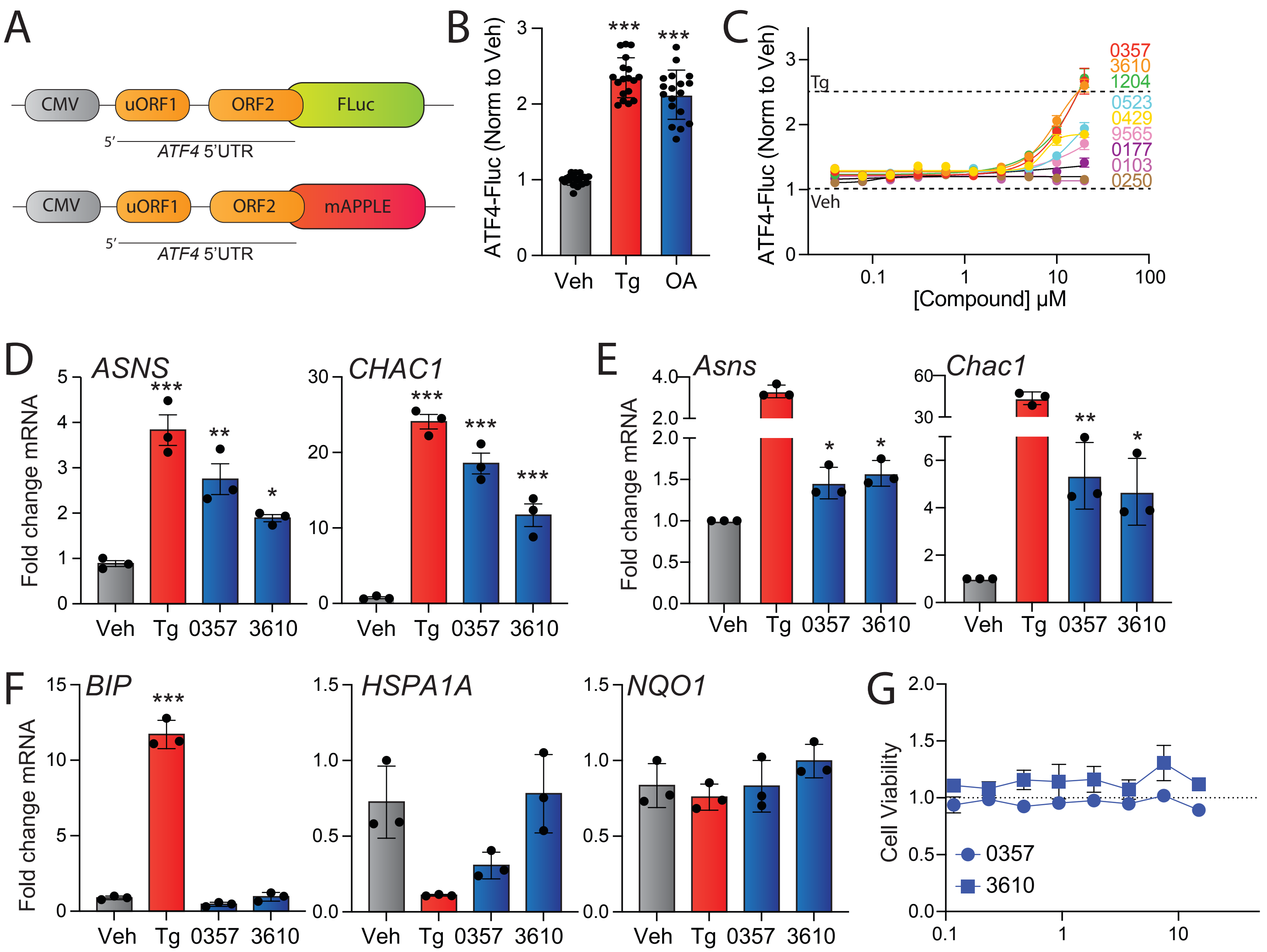

Figure S1 (Supplement to Figure 1). Identification of nucleoside mimetics that preferentially activate the ISR kinase HRI. **A.** The ATF4-FLuc and ATF4-mAPPLE reporters containing the 5'UTR of ATF4.[8,10] **B**. ATF4-Fluc activity, measured by luminescence, in HEK293 cells stably expressing ATF4-FLuc treated for 8 h with vehicle, thapsigargin (Tg, 0.5 μM), or oligomycin A (OA, 50 ng/mL). **C.** ATF4-FLuc activity, normalized to vehicle, in HEK293 cells stably expressing ATF4-FLuc treated for 8 h with the indicated dose of the indicated compound. The signals observed in veh or thapsigargin (Tg, 0.5 μM) cells is shown by the dashed lines. Error bars show SEM for n=9 replicates. **D**. Expression, measured by qPCR, of the ISR target genes *ASNS* and *CHAC1* in HEK293 cells treated for 8 h with vehicle, thapsgargin (Tg; 0.5 μM), 0357 (25 μM), or 3610 (25 μM). **E**. Expression, measured by qPCR, of the ISR target genes *Asns* and *Chac1* in MEF cells treated for 8 h with vehicle, thapsgargin (Tg; 0.5 μM), 0357 (20 μM), or 3610 (20 μM). **F**. Expression, measured by qPCR, of the UPR target gene *BiP*, the HSR target gene *HSPA1A*, and the OSR target gene *NQO1* in HEK293 cells treated for 8 h with vehicle, thapsgargin (Tg; 0.5 μM), 0357 (25 μM), or 3610 (25 μM). **G.** Viability, measured by Cell Titer Glo, of HEK293 cells treated for 24 h with the indicated concentration of 0357 or 3610. Error bars show SEM for n=3 replicates. *p<0.05, **p<0.01 ***p<0.005 for one-way ANOVA.

bioRxiv preprint doi: https://doi.org/10.1101/2024.06.10.598126; this version posted November 18, 2024. The copyright holder for this preprint (which was not certified by peer review) is the author/funder, who has granted bioRxiv a license to display the preprint in perpetuity. It is made available under aCC-BY 4.0 International license.

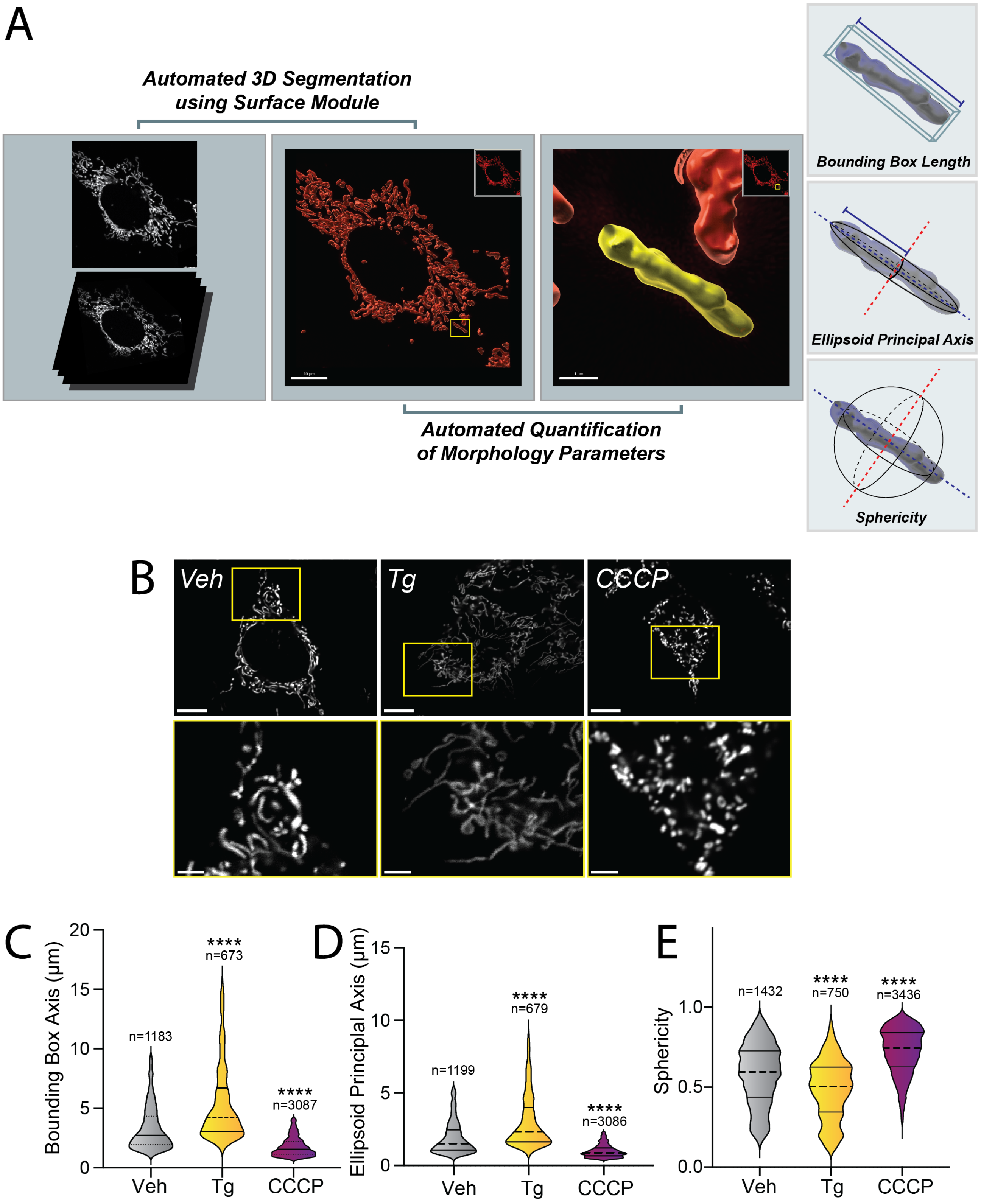

Figure S2 (Supplement Figure 2). Pharmacologic HRI activation induces ISR-dependent mitochondrial elongation. A. Image processing and analysis workflow to quantify several parameters that define mitochondrial shape. **B.** Representative images of MEF^{mtGFP} cells treated for 6 h with vehicle (veh), thapsigargin (Tg, 500 nM), or CCCP (10 μM). The inset shows a 3-fold magnification of the region indicated by the yellow box. Scale bars, 10 μm (top) and 3.33 μm (bottom) **C-E.** Quantification of bounding box axis, ellipsoid principal axis, and sphericity from the entire dataset of representative images shown in (**B**). The number of 3D segmentations used for the individual measurements for each condition are shown above. ****p<0.001 for Kruskal-Wallis ANOVA. Black asterisks show comparison with vehicle-treated cells.

bioRxiv preprint doi: https://doi.org/10.1101/2024.06.10.598126; this version posted November 18, 2024. The copyright holder for this preprint (which was not certified by peer review) is the author/funder, who has granted bioRxiv a license to display the preprint in perpetuity. It is made available under aCC-BY 4.0 International license.

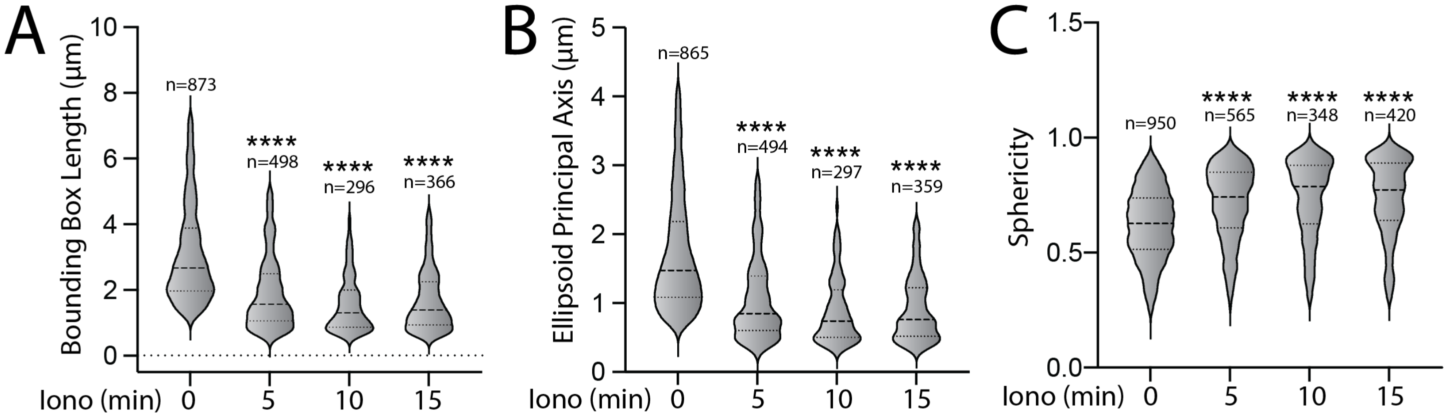

Figure S3. (Supplement to Figure 3). Pharmacologic activation of ISR kinases prevents ionomycin-dependent accumulation of fragmented mitochondria. A-C. Quantifications of bounding box axis, ellipsoid principal axis, and sphericity of MEF^{mtGFP} cells pre-treated for 6 h with vehicle and then challenged with ionomycin (1 µM) for the indicated time. Representative images are shown in **Fig. 3A**. The number of 3D segmentations used for the individual measurements for each condition are shown above. ****p<0.001 for Kruskal-Wallis ANOVA. Black asterisks show comparison with vehicle-treated cells at time 0.

bioRxiv preprint doi: https://doi.org/10.1101/2024.06.10.598126; this version posted November 18, 2024. The copyright holder for this preprint (which was not certified by peer review) is the author/funder, who has granted bioRxiv a license to display the preprint in perpetuity. It is made available under aCC-BY 4.0 International license.

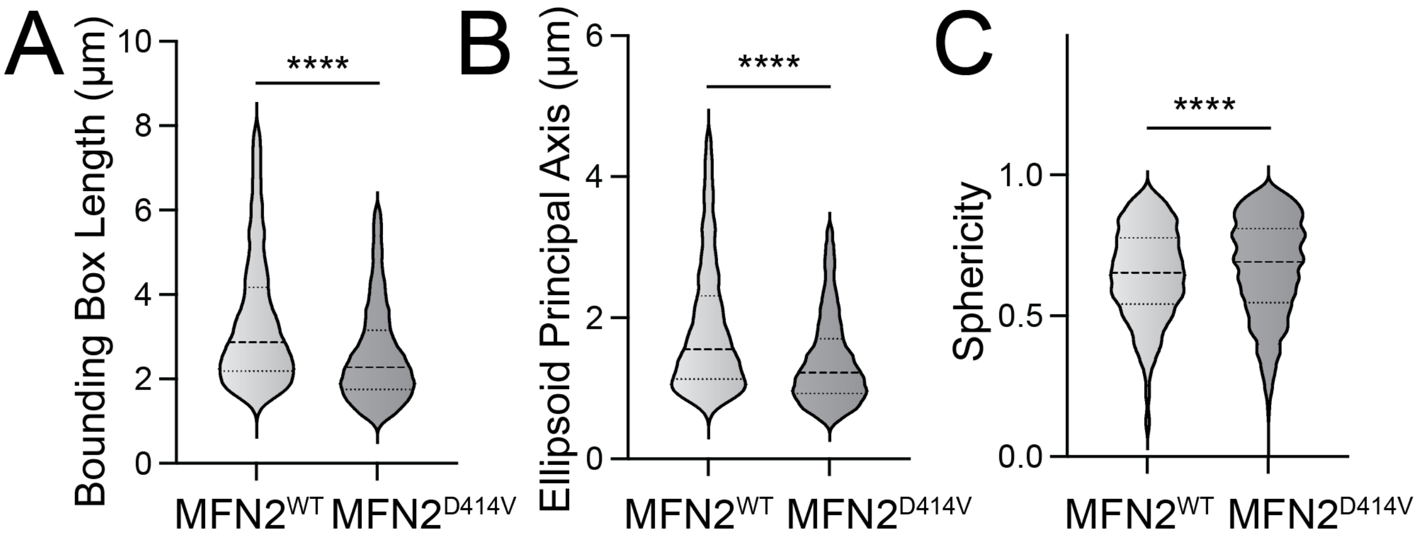

Figure S4 (Supplement to Figure 4). Pharmacologic activation of ISR kinases rescue basal mitochondrial morphology in patient fibroblasts expressing the disease-associated D414V MFN2 variant. A-C. Bounding box length (**A**), ellipsoid principal axis length (**B**), and sphericity (**C**) in control human fibroblasts expressing $MFN2^{WT}$ or patient fibroblasts expressing $MFN2^{D414V}$. Representative images are shown in **Fig. 4A,E**. ****$p<0.001$ for Mann-Whitney t-test.
